# Evaluation of the Sublimation Process of Some Purine Derivatives: Sublimation Rate, Activation Energy, Mass Transfer Coefficients and Phenomenological Models

**DOI:** 10.3390/ma15207376

**Published:** 2022-10-21

**Authors:** Cerasela-Ionela Cleminte, Daniela Ionita, Cătălin Lisa, Mariana Cristea, Ioan Mamaligă, Gabriela Lisa

**Affiliations:** 1 Faculty of Chemical Engineering and Environmental Protection “Cristofor Simionescu”, “Gheorghe Asachi” Technical University of Iasi-Romania, 73 D. Mangeron Street, 700050 Iasi, Romania; 2 “Petru Poni” Institute of Macromolecular Chemistry, Aleea Gr. Ghica Voda 41 A, 700487 Iasi, Romania

**Keywords:** caffeine, theophylline, sublimation, mass transfer, phenomenological models

## Abstract

Caffeine and theophylline are compounds with important applications in the pharmaceutical industry and other fields of the chemical industry. These purine derivatives have simple chemical structures, therefore, the evaluation of their sublimation process contributes to the development of mass transfer analysis methods that can later be applied to other compounds with more complex structures. With the help of thermogravimetric analysis in isothermal conditions, the kinetic study of the sublimation of caffeine and theophylline, along with the evaluation of kinetic parameters (activation energy and the pre-exponential factor), was carried out. Global mass transfer coefficients were determined, which vary for caffeine between 53 × 10^−8^ and 631 × 10^−8^ mol/s·m^2^·Pa, and for theophylline between 68 × 10^−8^ and 441 × 10^−8^ mol/s·m^2^·Pa. The dimensionless equations of the form: Sh=a+b·Rec·Scd have been proposed, which allow the determination of individual mass transfer coefficients at temperatures between 130 and 160 °C for caffeine and between 170 and 200 °C for theophylline.

## 1. Introduction

Purine derivatives like caffeine and theophylline are biologically active substances with important applications in the pharmaceutical industry [1]. There are also other applications for these compounds that have been identified by researchers in various fields of the chemical industry [1,2,3]. For example, caffeine has been shown to be a good inhibitor of the corrosion process of certain metals and alloys and can be used as an indicator in methods for detecting microbes in surface waters [1]. Wang et al. determined that caffeine can be used to improve the performance of perovskite metal halide solar cells [2]. Theophylline was used by H. Mohammed to obtain azo dyes of theophylline and their complexes with positive divalent cobalt and nickel ions [3].

Caffeine and theophylline have simple structures; therefore, the evaluation of their sublimation process is useful for the development of mass transfer analysis methods, which could later be applied to compounds with more complex structures.

The evaluation of mass transfer in the sublimation process requires accurate measurement of mass and temperature variations. Thermogravimetric analysis equipment allows the study of any physico-chemical process, provided that its development is accompanied by the variation of the mass of the system. Thermogravimetric analysis is used to study the sublimation process, due to the simplicity of the method and the accurate measurement of mass variation with temperature. Most of the existing literature studies the determination of vapor pressure, the evaluation of the sublimation enthalpy and the sublimation rate [4,5,6,7,8,9,10,11]. A Perkin-Elmer thermogravimetric analyzer was used by Dichi et al. to determine the vapor pressure and enthalpy of caffeine sublimation [4]. The same equipment was used by Soetaredjo et al. to analyze the sublimation process of (+)-catechin. The authors determined the vapor pressure at the following temperatures: 423, 428, 433, 438, 443K and calculated the sublimation enthalpy and entropy from the graphical representation ln(P) = f(1/T). They used anhydrous caffeine to calibrate the equipment. They determined that the sublimation rate of caffeine (dm/dt) was at the same temperature values shown previously (for which the vapor pressure was reported in the literature) and established the following equation: ln(P) = 1.135 × ln(dm/dt) + 0.225, which allowed the evaluation of the vapor pressure of (+)-catechin [5]. The rate of sublimation of solid crystalline 2-(2-nitrovinyl)furan was evaluated by Ruz et al. by isothermal thermogravimetric analysis in a TA-Instruments (Q600) to determine sublimation enthalpy and vapor pressure [6]. These authors used benzoic acid as a reference standard for vapor pressure estimation by thermogravimetric analysis with the following equation: ln(P) = 0.9146 × ln(dm/dt) + 2.1658 [6]. Ramos et al. [7] determined the enthalpy of sublimation for a series of organic compounds including of a group of cyclic ureas using a TA Instruments (New Castle, DE, USA) Q500 thermogravimetric analysis device. The authors determined that sublimation enthalpy increases only by 0.9–2.5 kJ/mol if the diffusional factor is taken into account. The values obtained for the sublimation enthalpies of the analyzed organic compounds are in full agreement with those reported in the literature, but using other experimental methods. Compared to other conventional techniques used to determine sublimation enthalpy, thermogravimetric analysis has the advantage of shorter experiments and small samples [7]. Karakaya et al. used thermogravimetric analysis to determine sublimation enthalpy and vapor pressure within the 570–640 K temperature range of the inorganic compound InCl_3_ using CuCl as a reference material [8]. Thermogravimetric analysis under isothermal conditions was also applied by Shahbazi et al. to determine sublimation enthalpy for a series of metal b-diketonate complexes [9]. Flores et al. [10] demonstrated that the application of thermogravimetric analysis under isothermal conditions is a reliable method for evaluating the enthalpy of sublimation. The additional enthalpy values for anthracene, pyrene and benzoic acid are comparable to those evaluated by other methods, with the uncertainty being ±2.2 kJ·mol^−1^. Using these substances as reference standards, the authors determined the enthalpy of sublimation for a family of flavones found in fruits, vegetables and various beverages, namely 3-, 5-, 6-, 7-hydroxyflavone and 6-aminoflavone [10]. Good reproducibility of sublimation enthalpy determined by isothermal thermogravimetric analysis for a series of metallocenes was also reported by Vieyra-Eusebio and Aaron Rojas [11].

In this study, for the first time, the use of thermogravimetric analysis techniques was extended for the evaluation of the mass transfer process in sublimation. This means the determination of the sublimation rate, the activation energy, the global mass transfer coefficients and the design of a phenomenological model. The model allows for the determination of individual mass transfer coefficients for caffeine and theophylline. Due to the simple structure of caffeine and theophylline, the evaluation of the sublimation process allows for the development of mass transfer analysis methods that may be used in the future for compounds with more complex structures.

## 2. Materials and Methods

All experimental tests were performed with commercial samples of caffeine and theophylline, as anhydrous powders (Purity 99%, Sigma-Aldrich, St. Louis, MO, USA). Table 1 shows the optimized chemical structures and some properties of these compounds. The geometry optimization of caffeine and theophylline molecules was performed with the software HyperChem 7.5. (Hypercube Inc., Gainesville, FL, USA) using the MM^+^ method, which is a common computational method in molecular mechanics that is particularly useful for organic molecules. The optimization algorithm selected was Polak–Ribière (conjugate gradient), with which the structures with minimum energy and minimum atomic forces were established.

The thermal behavior of caffeine and theophylline was analyzed in an ambient air atmosphere with a flow rate of 20 mL/min using Discovery TGA 5500 equipment (TA Instruments). This equipment provides a mass measurement accuracy of ±0.01% and a resolution of <0.1 μg. The samples, with mass between 5 and 5.5 mg, were heated at a rate of 10 °C/min in the temperature range 30–600 °C.

The sublimation process of caffeine and theophylline was also evaluated. The tests were carried out for one hour under constant temperature conditions: 130, 140, 150 and 160 °C for caffeine and 170, 180, 190 and 200 °C for theophylline. The mass loss was measured under constant temperature conditions in an ambient air atmosphere with a flow rate of 20 mL/min. Until a constant temperature was reached, the samples were heated from a temperature of 30 °C at a rate of 10 °C/min. Evaluation of the sublimation process by thermogravimetric analysis under isothermal conditions involved the use of small sample amounts: 4.6–5.1 mg of caffeine and 4.7–4.9 mg of theophylline.

The establishment of the phenomenological model for the sublimation of caffeine and theophylline in the crucible of the TGA, which provides a flat surface in contact with the entrainer (ambient air), was carried out with the help of programs made in Mathcad (MathSoft Engineering and Education, Inc., Cambridge, MA, USA).

The rate of sublimation is constant, at constant temperature, Equation (1):

r_subl_ = −dm/dt = k (1)

The temperature dependence of the sublimation rate constant can be described by an Arrhenius-type equation:

K = A e^−Ea/RT^ (2)

where A is the pre-exponential factor, Ea is the activation energy, R is the universal gas constant and T is the temperature expressed in K.

Sublimation kinetics of caffeine and theophylline were estimated using the logarithmic form of the Arrhenius equation:

ln(dm/dt) = lnA − E_a_/RT (3)

where dm/dt was the sublimation rate constant.

The thermogravimetric curves recorded in isothermal conditions allowed the study of the mass transfer process in sublimation.

With Equation (4) the global mass transfer coefficients for various temperatures were calculated.

(4) $$N_{A}=K_{p}\cdot S\cdot \Delta P_{\mathrm{med}}$$

where N_A_ was the amount of caffeine and theophylline sublimating in one second [mol/s], K_P_ was the global mass transfer coefficient [mol/s·m^2^·Pa], S was the surface of the crucible, which was 0.0000739 m^2^, and ∆P_med_ was the mass transfer potential in terms of pressure [Pa].

The mass transfer potential ∆P_med_ can be calculated as the difference between the vapor pressure of purine derivatives on the surface of the sample layer in the crucible and the vapor pressure of the caffeine or theophylline in the gaseous entrainer (ambient air).

The vapor pressure of caffeine at the surface of the layer, expressed in Pa, was calculated with the relation (5), and the vapor pressure of theophylline was calculated with the relation (6) [12]:

(5) $$\log\left(p_{v}\right)\left[\mathrm{Pa}\right]=-\frac{5477}{T\left[K\right]}+14.395$$

(6) $$\log\left(p_{v}\right)\left[\mathrm{Pa}\right]=-\frac{6896}{T\left[K\right]}+16.027$$

The establishment of the phenomenological model for the sublimation of purine derivatives from the crucible of the Discovery TGA 5500 (TA Instruments), which ensures a flat surface in contact with the entrainer (air), was carried out with the help of programs made in Mathcad. The dimensionless equation used to determine the individual mass transfer coefficient $k_{g}$ has the form:

(7) Sh=a+b·Rec·Scd

The Sherwood number has the following mathematical expression:

(8) $$\mathrm{Sh}=\frac{k_{g}\cdot h_{P}}{D_{p}}$$

where:

$k_{g}$ is the individual mass transfer coefficient (m^3^/m^2^·s);
h_p_ is the height of the purine derivatives layer (m);
D_p_ is the diffusion coefficient of purine derivative vapors in air for which values from the literature were used (m^2^/s) [13].

The following mathematical expression was used for the global coefficient K_p,_ which allowed the calculation of the individual mass transfer coefficient:

(9) $$K_{p}=k_{g}\frac{p}{R\cdot T}$$

where:

P—working pressure in the equipment, which was assumed to be atmospheric pressure (1.013 × 10^5^ Pa);
R—universal gas constant (8.314 J/mol·K);
T—temperature of the driving gas expressed in K.

The Reynolds number was calculated with the following relation:

(10) Re=ρair·vm·hpηair

where:

$\rho_{\mathrm{air}}$—density of the entrainer calculated at the working temperature;
$\eta_{\mathrm{air}}$—viscosity of the entrainer calculated at working temperature;
$v_{m}$—the rate of the entrainer (0.003 m/s).

The Schmidt number was calculated with the following relation:

(11) $$\mathrm{Sc}=\frac{\eta_{\mathrm{air}}}{\rho_{\mathrm{air}}\cdot D_{p}}$$

for which the meaning of the sizes has been previously presented. The Schmidt number is a property of the fluid (air) that depends on the working temperature, not on the sample size. This is why it was considered at a power that did not change in the model, namely 0.33. The same value was also obtained for the coefficient c, the power to which the Reynolds number was raised. This number included the height of the layer of purine derivatives h(m). However, it did not vary significantly at the temperatures at which the experimental determinations were carried out.

## 3. Results and Discussion

### 3.1. Thermal Behavior

**Figure 1:**
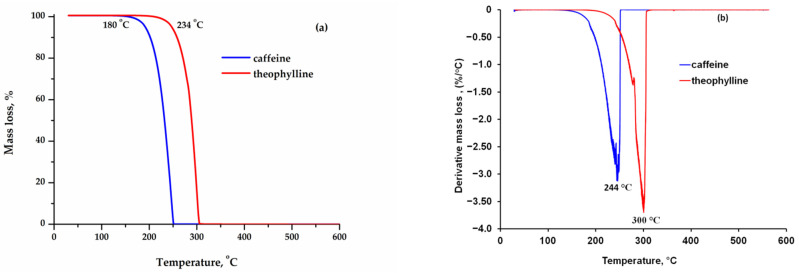
TG (**a**) and DTG (**b**) curves.

The evaluation of the thermal behavior was carried out in an ambient air atmosphere with a rate of 10°C/min. Thermogravimetric (TG) and derivative thermogravimetric (DTG) curves are shown comparatively in Figure 1a,b. The mass loss was found to occur completely in one step between 180–251 °C for caffeine and 234–305 °C for theophylline. It was found that the mass loss of both samples occurs within a temperature range of 71 °C. The temperature at which the rate of the mass loss reaches its maximum is 244 °C for caffeine and 300 °C for theophylline. Most studies in the literature investigate the thermal behavior for purine derivatives in an inert atmosphere (nitrogen, helium) and at various heating rates: 3, 5 și 20 °C/min [4,14,15,16]. M. Wesolowski and P. Szynkaruk analyzed the thermal behavior in ambient air atmosphere for samples of caffeine and theophylline with a mass of 50, 100 and 200 mg at the following heating rates: 3, 5, 10 and 15 °C/min. They established that the thermal behavior was influenced both by the mass of the samples in the study and by the heating rate [17,18].

Circioban D. et al. evaluated the thermal behavior in air for 5 mg of theophylline with a heating rate of 10 °C/min and found, as in this study, a complete mass loss in a single step between 217–322 °C, with the temperature at which the mass loss rate was maximum being 319 °C [19]. With equipment similar to that used in this study, B. Rojek and M. Wesolowski [20] evaluated theophylline thermal behavior in air with the following heating rates: 2.5, 5, 10 and 20 °C/min and an approximately 10 mg sample. The results established for the rate of 10 °C/min were very close to those obtained in this study, namely a complete mass loss for theophylline in a single step in the temperature range between 225–295 °C. For caffeine, Wang et al. [2] reported the mass loss in air at a heating rate of 10 °C/min and a single step in the temperature range 200–285 °C, but without specifying the amount of sample used.

**Figure 2:**
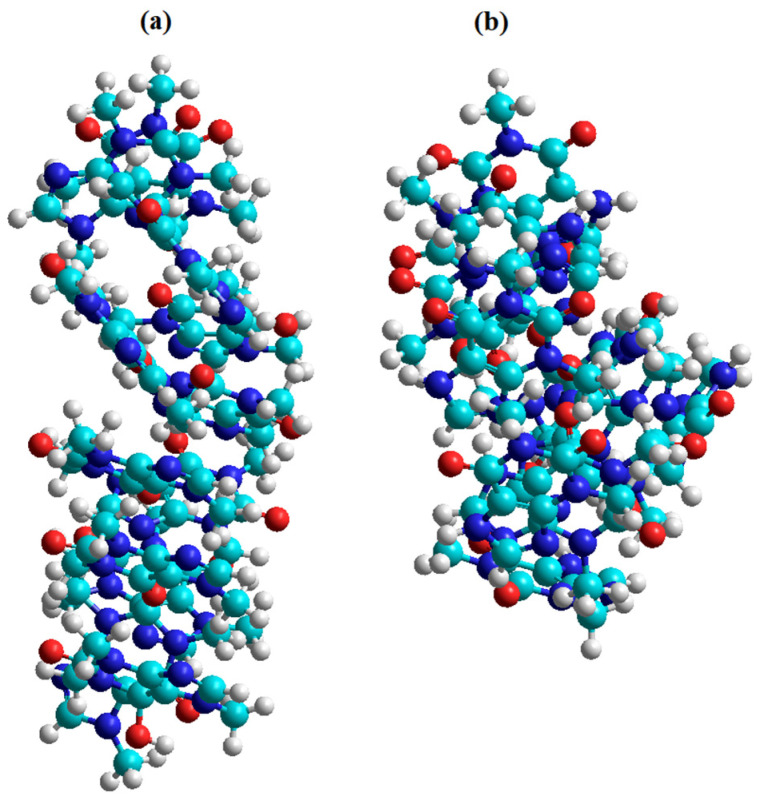
Optimized geometry representing ball shape for 12 caffeine molecules (**a**) and 12 theophylline molecules (**b**).

The lower thermal stability of caffeine compared to theophylline is also suggested by molecular modeling with HyperChem software. If 12 caffeine and theophylline molecules were considered and the Polak–Ribière optimization algorithm was used, stronger interactions were found in the theophylline molecules than in the caffeine molecules. According to the results obtained and represented in Figure 2, the organization of the 12 theophylline molecules was more compact than in the caffeine molecules. The total energy obtained for the theophylline molecules at a temperature of 430 K was 3,429,415.6 J/mol, and for caffeine was 3,827,021.12 J/mol, suggesting that caffeine is less stable. The conformation with the lowest energy is considered the most stable [21].

### 3.2. Kinetic Study of the Sublimation of Purine Derivatives

In general, the sublimation process involves the following steps:

(1) The transfer of thermal energy in the solid;
(2) The breaking of intermolecular bonds in the crystal lattice, leading to the formation of free molecules;
(3) Vapor transport to the surface and its entrainment.

The kinetics of sublimation can be controlled by the rate of any of these processes. In the case of the sublimation process evaluated in this study, we consider that the determinant rate is the second stage in which the breaking of the intermolecular bonds in the crystalline network takes place [22]. Evaluation of the sublimation process by thermogravimetric analysis under isothermal conditions involved the use of small amounts of sample: 4.6–5.1 mg caffeine and 4.7–4.9 mg theophylline. Considering that small amounts of sample were used, it can be assumed that heat transfer to the powder sample was fast enough. The temperatures at which the thermogravimetric curves were recorded, under constant temperature conditions, were selected by analyzing the thermal behavior of the samples when the temperature increased at a 10 °C/min heating rate, as well as considering the temperature ranges for which vapor pressure data for these compounds were available in the literature.

**Figure 3:**
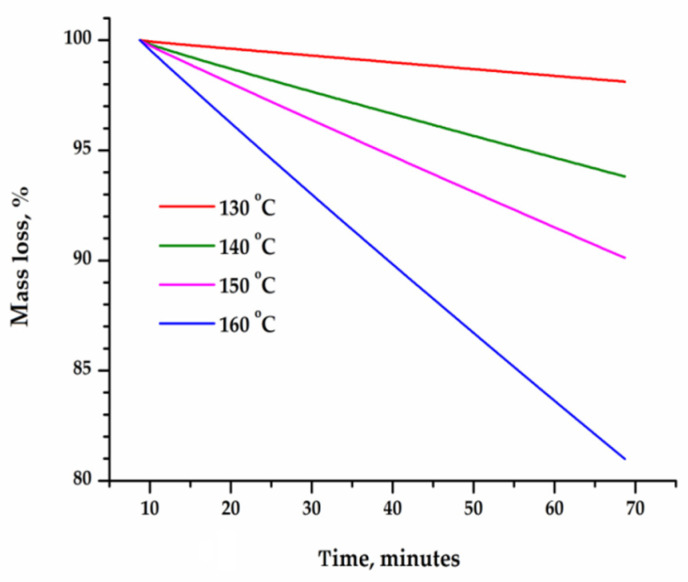
Percentage loss of mass over time for the sublimation of caffeine at various temperatures: 130, 140, 150 and 160 °C.

**Figure 4:**
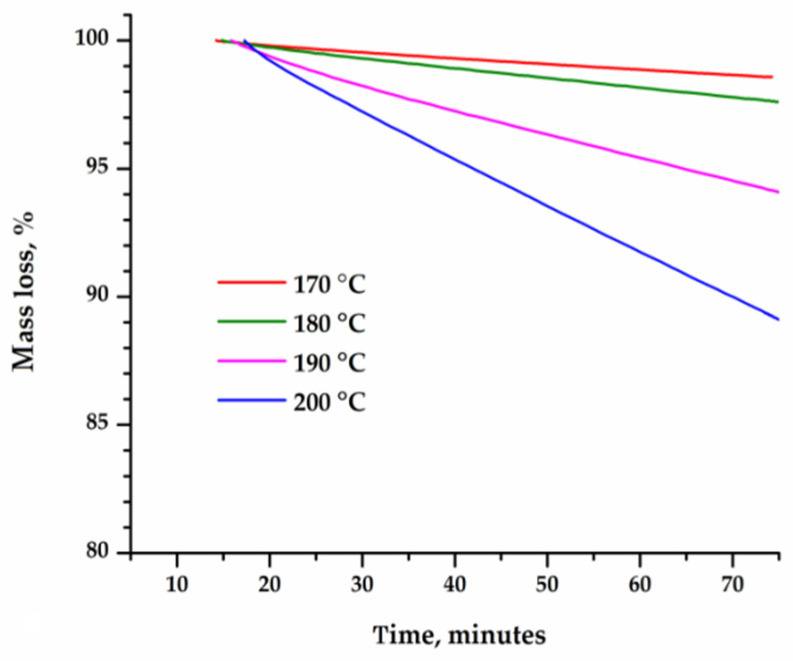
Percentage loss of mass over time for the sublimation of theophylline at various temperatures: 170, 180, 190 and 200 °C.

**Figure 5:**
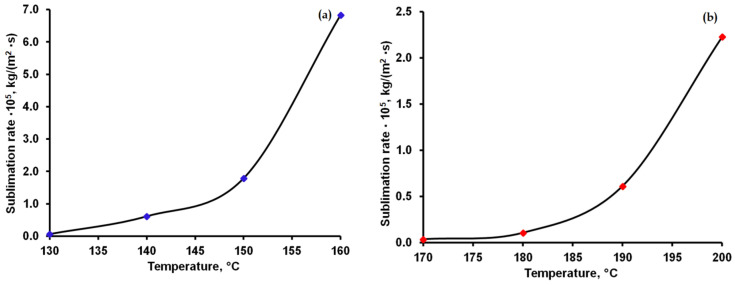
The variation of the sublimation rate depending on the temperature for caffeine (**a**) and theophylline (**b**).

The time dependence of the change in the mass of sublimated caffeine and theophylline (Figure 3 and Figure 4) was linear at each temperature, which indicates that the sublimation process follows a zero-order kinetics. The rate of sublimation, calculated on the basis of thermogravimetric curves recorded in isothermal conditions, increased exponentially with temperature, as seen in Figure 5.

**Figure 6:**
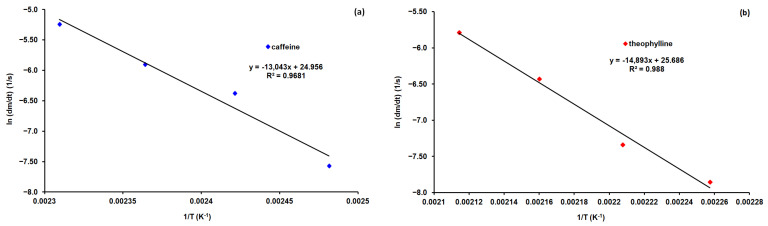
Calculation of the parameters of the Arrhenius equation for caffeine (**a**) and theophylline (**b**).

Graphically, representing ln(dm/dt) as a function of 1/T (Equation (3)), linear dependencies were obtained, as can be seen in Figure 6. The activation energy was calculated from the slope of the lines obtained, and the pre-exponential factor was obtained from the intercept at the origin. The results obtained are presented in Table 2.

The kinetic study of the sublimation of caffeine and theophylline, carried out with the help of thermogravimetric analysis in isothermal conditions, allowed the evaluation of the kinetic parameters. The activation energy and the pre-exponential factor were higher for theophylline compared to caffeine (Table 2). The rate of mass loss followed zero-order kinetics. The sublimation rate of caffeine was several times higher compared to that of theophylline. F.E. Soetaredjo et al. [5] carried out a kinetic study of (+)-catechin hydrate sublimation using thermogravimetric analysis at a constant temperature. Through this method, they determined the rate of sublimation (dm/dt) at various temperatures. From the logarithmic form of the Arrhenius equation, they obtained for the activation energy 194.43 kJ/mol and the pre-exponential factor 54.238. No studies that provided information on the sublimation kinetics of caffeine and theophylline were identified. Buzyurov et al. recently used a fast-scanning calorimeter to determine the vapor pressure and sublimation enthalpy for a series of compounds, including caffeine and theophylline. Experimental determinations were made within the 131–211 °C temperature range for caffeine and 181–241 °C temperature range for theophylline. Sublimation enthalpy determined by this experimental technique was 110 kJ/mol for caffeine and 130 kJ/mol for theophylline [23].

### 3.3. Evaluation of Global Mass Transfer Coefficients

In the gaseous entrainer, the vapor pressure for purine derivatives has a small value, which at the limit can be considered zero. Table 3 shows the mass transfer potential values as a function of temperature.

**Figure 7:**
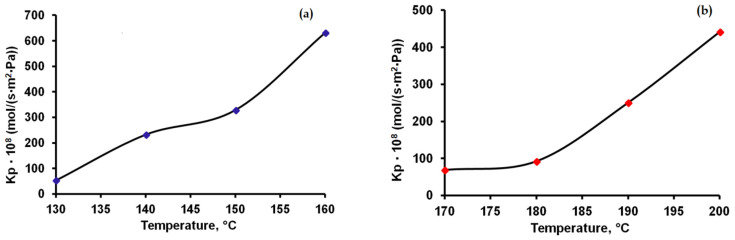
Influence of temperature on the mass transfer process for caffeine (**a**) and theophylline (**b**).

Knowing the sublimation rate expressed in kg/m^2^·s, calculated and shown in Figure 5, the molecular mass of caffeine 194.19 g/mol, the molecular mass of theophylline 180.17 g/mol and the mass transfer potential values shown in Table 3, the global mass transfer coefficients K_P_ expressed in mol/s·m^2^·Pa were calculated at various temperatures. The results obtained are shown in Figure 7a,b. The overall mass transfer coefficients for caffeine were higher than for theophylline. In the case of theophylline, a sharper increase in the global transfer coefficients was observed at temperatures higher than 180 °C, while for caffeine the curve showed an inflection point in the temperature range between 140–150 °C.

### 3.4. Establishing the Phenomenological Model

**Figure 8:**
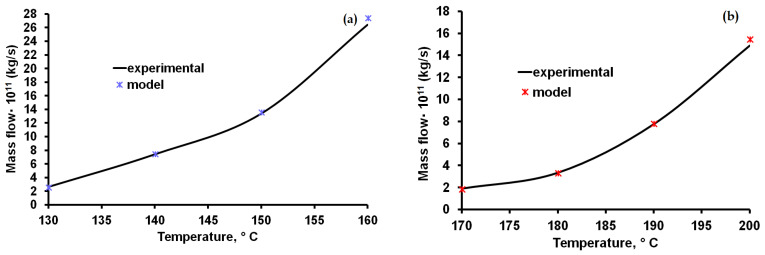
Influence of temperature on the mass flow of sublimated caffeine (**a**) and theophylline (**b**).

The values of the constants a, b, c and d for the phenomenological model (Equation (7)), presented in Table 4, were established through successive tests until the sublimating mass flow, expressed in kg/s, approached the value obtained experimentally (Figure 8).

The obtained dimensionless equations made possible the determination of the individual mass transfer coefficients for the sublimation of caffeine at temperatures between 130 and 160 °C and between 170 and 200 °C for theophylline. According to the graphic representation presented in Figure 8, there were greater deviations of the phenomenological model compared to the experimental data at higher temperature values. For caffeine, the deviation is 3.8% at 160 °C and is below 1.6% at lower temperatures. In the case of theophylline, the deviation is 4.1% at 200 °C, and similarly, goes down at lower temperatures to below 1.2 %. Mass transfer coefficients for caffeine and theophylline were determined by Abdelaziz et al. [24] in their study of the sublimation process that used the fast-scanning calorimetry (FSC) technique. The studies carried out by these researchers allowed the experimental determination of the vapor pressure and the enthalpy of sublimation in a temperature range between 442–538 K for theophylline and 395–410 K for caffeine. For a temperature of 510K, the average sublimation rate reported by Abdelaziz et al. [24] for theophylline in the first 5 s after the start of the experiment was approximately 3 g/m^2^·s. If we extrapolate the experimental results from our study for a temperature of 510 K, the resulting sublimation rate is 3.67 g/m^2^·s. Also, the initial mass flow reported for theophylline at 360 K by Abdelaziz et al. [24] was 1.7·10−11 kg/s, and in our study, at the lowest temperature value at which experimental determinations were made, was 2·10−11 kg/s.

## 4. Conclusions

The thermal stability of caffeine and theophylline was evaluated under dynamic and isothermal conditions in air with a Discovery TGA 5500 (TA Instruments). Thermal behavior in air at a heating rate of 10 °C/min occurred completely, in one step, within the temperature range of 180–251 °C for caffeine and 234–305 °C for theophylline. The time dependence of the mass change of sublimated caffeine and theophylline was linear at constant temperature, indicating that the sublimation process follows zero-order kinetics. The rate of sublimation increased exponentially with temperature. This varied between 0.06 × 10^−5^ and 6.83 × 10^−5^ kg/m^2^·s for caffeine and between 0.03 × 10^−5^ and 2.23 × 10^−5^ kg/m^2^·s for theophylline. The rate of sublimation was several times higher in caffeine than in theophylline.

The kinetic study of the sublimation of caffeine and theophylline, carried out with the help of thermogravimetric analysis in isothermal conditions, allowed the evaluation of the kinetic parameters. The value of activation energy and pre-exponential factor were higher for theophylline than for caffeine.

The global mass transfer coefficients varied between 53 × 10^−10^ and 631 × 10^−10^ (mol/s·m^2^·Pa) for caffeine and between 68 × 10^−10^ and 441 × 10^−10^ (mol/s·m^2^·Pa) for theophylline.

The obtained dimensionless equations enabled the determination of individual mass transfer coefficients for the sublimation of caffeine and theophylline. This research may be extended in the future to evaluate mass transfer in the sublimation process of some compounds with more complex structures. The chemical vapor deposition (CVD) technique and its variants are extremely important from an economic point of view as they enable the manufacture of nanomaterials, nanostructured films and composite coatings. Their development is closely related to the study of the sublimation process and of the modeling and optimization of the mass transfer process in sublimation.

## Figures and Tables

**Table 1:** Optimized molecular structures representing ball shape and their properties.

Caffeine	Theophylline
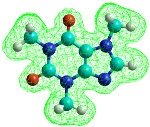	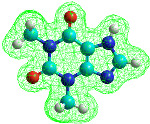
Molecular mass = 194.19 g/mol Surface = 361.94 Å^2^ (grid) Volume = 568.14 Å^3^ Hydration energy = −9372.16 J/mol Total energy = 11,731.94 J/mol RMS gradient = 320.75 J/Å·mol	Molecular mass = 180.17 g/mol Surface = 336.99 Å^2^ (grid) Volume = 518.04 Å^3^ Hydration energy = −22,468.08 J/mol Total energy = 92,675.6 J/mol RMS gradient = 306.65 J/Å·mol

Cyan is carbon, red is oxygen, nitrogen is blue and white is hydrogen.

**Table 2:** Kinetic parameters from the Arrhenius equation.

Substance	Caffeine	Theophylline
Ea, kJ/mol	108.439	123.820
lnA	24.956	25.686
r^2^	0.9681	0.9880

**Table 3:** Mass transfer potential values at various temperatures.

Caffeine		Theophylline	
Temperatura, [°C]	$\Delta P_{\mathrm{med}},\;[\mathrm{Pa}]$	Temperatura, [°C]	$\Delta P_{\mathrm{med}},\;[\mathrm{Pa}]$
130	6.374	170	2.887
140	13.599	180	6.368
150	27.990	190	13.578
160	55.724	200	28.036

**Table 4:** The values of coefficients a, b, c, d in the dimensionless Equation (7).

Sample	Temperature (⸰C)	Model Parameters			
		a	b	c	d
Caffeine	130	49.5	1	0.66	0.33
	140	119	1	0.66	0.33
	150	240	1	0.66	0.33
	160	482	1	0.66	0.33
Theophylline	170	29	1	0.66	0.33
	180	53	1	0.66	0.33
	190	114.6	1	0.66	0.33
	200	222	1	0.66	0.33
